# Signatures of Amorphous Shiba State in FeTe_0.55_Se_0.45_

**DOI:** 10.1021/acs.nanolett.4c05650

**Published:** 2025-02-12

**Authors:** Jinwon Lee, Sanghun Lee, Andreas Kreisel, Jens Paaske, Brian M. Andersen, Koen M. Bastiaans, Damianos Chatzopoulos, Genda Gu, Doohee Cho, Milan P. Allan

**Affiliations:** † Leiden Institute of Physics, Leiden University, Leiden 2333CA, The Netherlands; § Department of Physics, Yonsei University, Seoul 03722, Republic of Korea; ⊥ Center for Quantum Devices, Niels Bohr Institute, University of Copenhagen, Copenhagen Ø 2100, Denmark; # Condensed Matter Physics and Materials Science Department, Brookhaven National Laboratory, Upton, New York 11973, United States; ∇ Faculty of Physics, Ludwig-Maximilians-University Munich, Munich 80799, Germany; ○ Center for Nano Science (CeNS), Ludwig-Maximilians-University Munich, Munich 80799, Germany; ◆ Munich Center for Quantum Science and Technology (MCQST), Ludwig-Maximilians-University Munich, Munich 80799, Germany

**Keywords:** Iron-based superconductor, FeTe_0.55_Se_0.45_, Shiba states, Scanning tunneling microscopy and spectroscopy

## Abstract

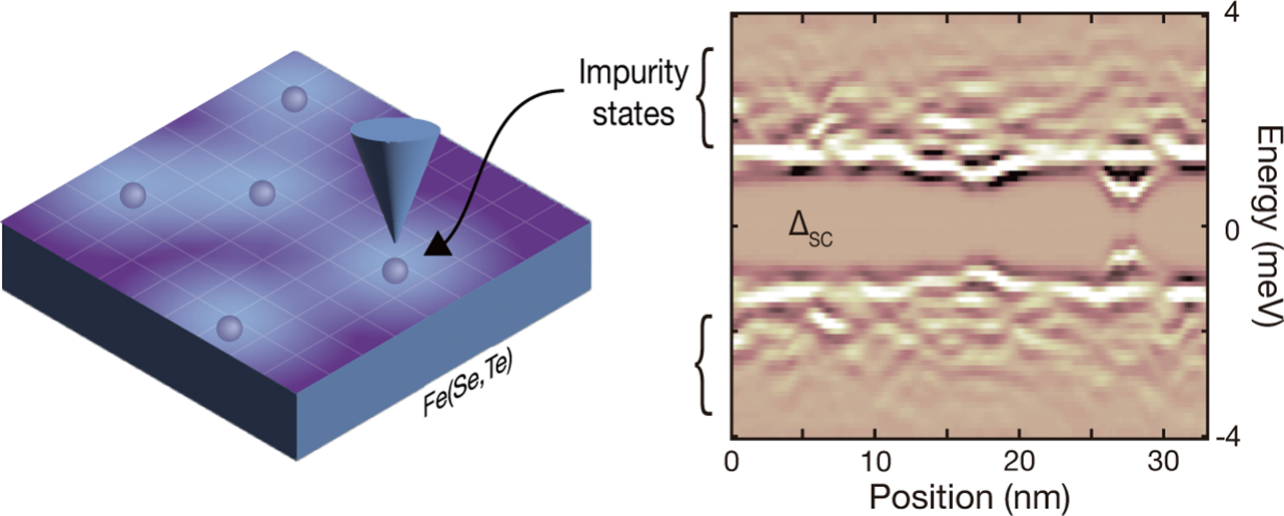

The iron-based superconductor FeTe_0.55_Se_0.45_ is a peculiar material: it hosts surface states with a Dirac dispersion, is a putative topological superconductor hosting Majorana modes in vortices, and has an unusually low Fermi energy. The superconducting state is generally characterized by three gaps in different bands, with the homogeneous, spatially extended Bogoliubov excitations—in this work, we uncover evidence that it is instead of a very different nature. Our scanning tunneling spectroscopy data show several peaks in the density of states above a full gap, and by analyzing their spatial and junction-resistance dependence, we conclude the peaks above the first one are not coherence peaks from different bands. Instead, comparisons with our simulations indicate they originate from generalized Shiba states that are spatially overlapping. This can lead to an amorphous state of Bogoliubov quasiparticles, reminiscent of impurity bands in semiconductors. We discuss the origin and implications of this new state.

In the superconducting state, FeTe_0.55_Se_0.45_ exhibits a number of unusual properties .^1−7^ It is highly disordered, basically being an alloy of FeSe and FeTe (Figure 1a), and it remains to be resolved exactly how much remnant magnetism exists. Despite this, superconductivity is robust, with a transition temperature *T*_c_ of 14.5 K. The pairing symmetry is widely believed to be s_±_^2,3,8^ with different gap sizes on different bands/orbitals. There are two hole-pockets at the Γ point and two electron pockets at the M point; the smaller (larger) hole pocket is referred to as α′ (β), and the electron pocket γ band (Figure 1b). Recent angle-resolved photoemission spectroscopy (ARPES) studies revealed that the α′, β, and γ bands have a superconducting gap with energy values of roughly 1.4, 2.4, and 4.5 meV, respectively.^9^ One would expect that scanning tunneling microscopy (STM) and spectroscopy reflect the multiple-gap structure determined by several coherence peaks similar to other multiband superconductors like LiFeAs^10^ or NbSe_2_:^11^ two sets of sharp coherence peaks with the larger gapping out a proportion of the spectral weight, and the smaller one the rest. Previous STM data on FeTe_0.55_Se_0.45_, taken with less energy resolution, appeared to show just one single gap of 1.7 meV.^3^ However, better energy resolution revealed a much more complex set of peaks, reminiscent of—and previously identified as—coherence peaks of the Bogoliubov density of states (DOS) from the different bands.^12,13^ It was conjectured that the significant spatial variations of the spectral features are due to disorder causing electronic heterogeneity.^14^

**Figure 1:**
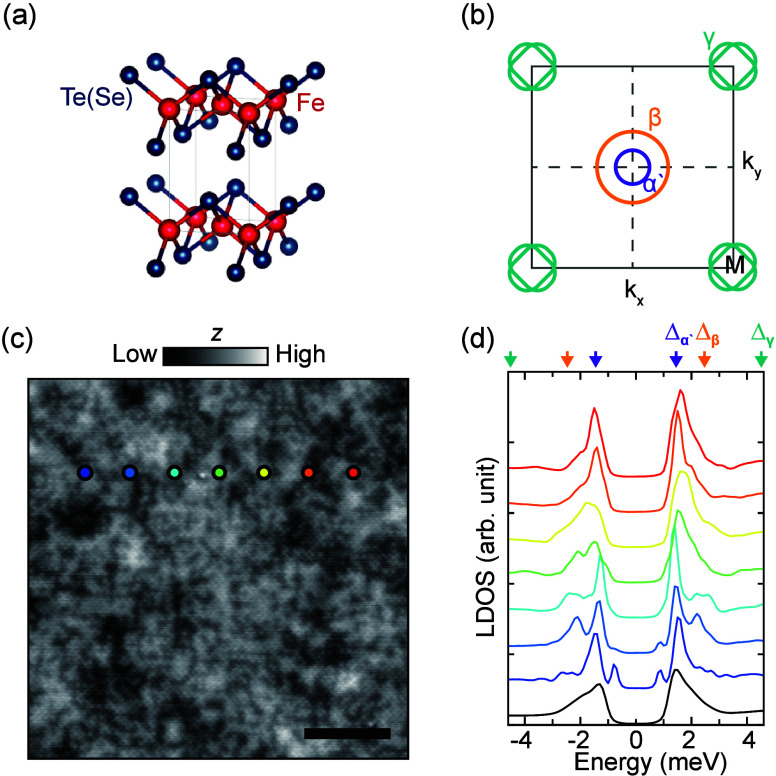
Disordered superconductor FeTe_0.55_Se_0.45_. (a) Schematic of the FeTe_0.55_Se_0.45_ crystal structure. (b) Fermi contours of FeTe_0.55_Se_0.45_. Two hole-like bands (α′ and β) at the center of the Brillouin zone and the folded electron-like band (γ) at the corners of the Brillouin zone. (c) STM topography of FeTe_0.55_Se_0.45_ taken with *V*_set_ = −6 mV and *I*_set_ = 120 pA. Scale bar, 5 nm. (d) Differential conductance (d*I*/d*V*) measured at the sites marked by colored dots in panel c. The black curve is a spatially averaged spectrum. Each curve is shifted for clarity. The colored arrows indicate the multiple superconducting gaps assigned by previous ARPES measurements.^9^

In this Letter, we demonstrate that our high-energy-resolution spectroscopic data is inconsistent with an interpretation of multiple coherence peaks. The local density of states (LDOS) mapping reveals that the observed peaks are unlike a single coherence peak but spatially dispersive and influenced by the electric field of the tip, similar to low-energy Shiba states.^15^ These experimental observations let us exclude the possibility that the spectral peaks beyond the first ones are the coherence peaks of the larger superconducting gaps. Instead, based on good agreement with simulations, we argue that these peaks are generalized Shiba states associated with a larger gap. “Shiba states” usually denote the low-lying excitations, resulting from paramagnetic impurities in s-wave superconductors; here we use the term generalized Shiba state^16^ for in-gap states, independent of whether they originate from nonmagnetic or magnetic impurities, as the effect is similar in superconductors with sign-changing gaps.^17−19^ They are randomly distributed, and might be caused by the random distribution of the two different chalcogen atoms,^14,20,21^ or the remnant nonperiodic spin structure in the iron lattice.^22^ Either can produce a landscape with emergent locations favorable for generating generalized Shiba states. These states have substantial spatial overlap and are likely to form an amorphous quasiparticle state below the largest superconducting gap. The ramification of this scenario is that the low-energy electronic properties of FeTe_0.55_Se_0.45_ are characterized by an amorphous Shiba state.

To arrive at these findings, we cleave FeTe_0.55_Se_0.45_ crystals^23^ in an ultrahigh vacuum at about 30 K and perform spectroscopic-imaging STM experiments at 2.2 K. For increased energy resolution, we use a tip with a superconducting apex with a gap of Δ_tip_ = 1.3 meV and a broadening of Γ = 45 μeV.^24^ The spectra shown in Figures 1–3 are deconvoluted using the tip spectrum obtained from experiments on a Pb(111) surface as described in ref.^15^ (see Supporting Information Figures S1–S3 for non-deconvoluted spectra). To understand the observations theoretically, we start from a multiband model of the electronic structure, include a superconducting interband and intraband order parameter as expected from spin-fluctuation pairing, and calculate the LDOS by including disorder within a T-matrix approach (see Supporting Information for more details).

**Figure 2:**
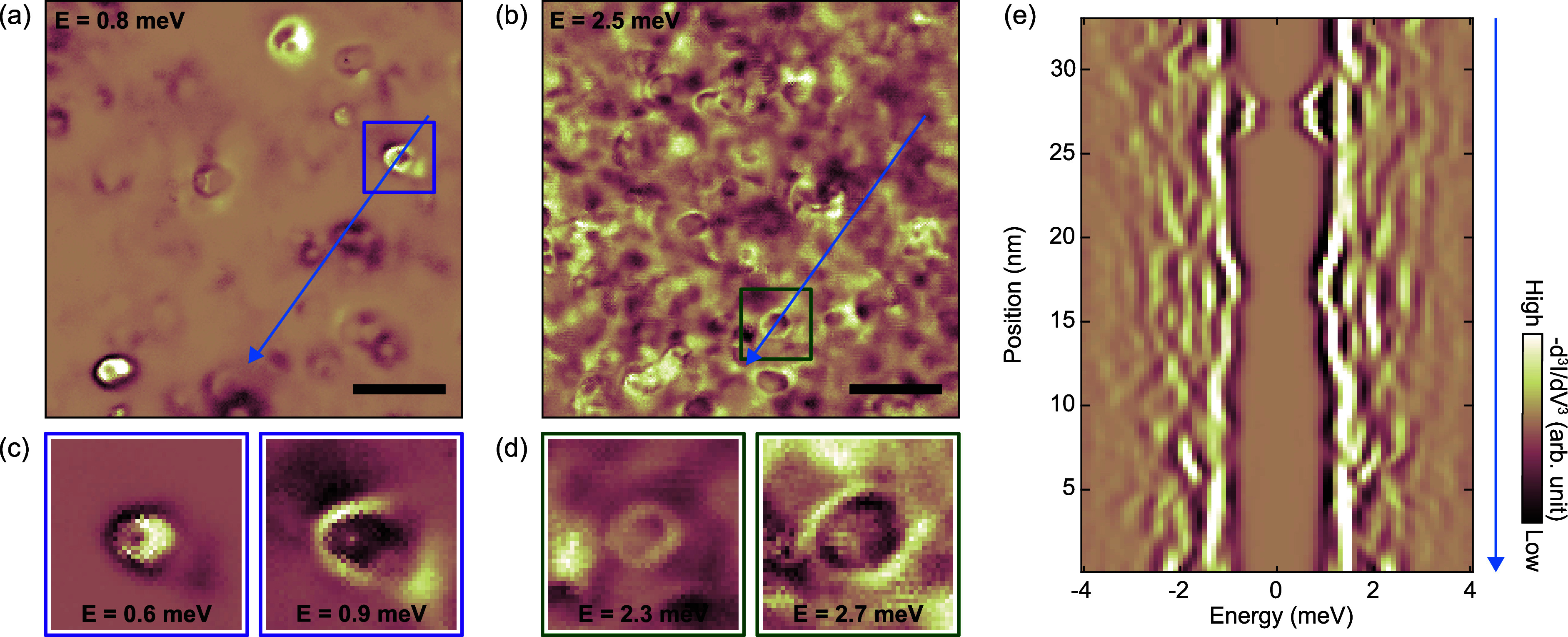
Spatially dispersive generalized Shiba states inside and outside the superconducting gap. (a and b) Negative second derivative of deconvoluted differential conductance (−d^3^*I*/d*V*^3^) maps at the energies of +0.8 and +2.5 meV for the same field of view. The d*I*/d*V* maps are acquired with *V*_set_ = −6 mV, *I*_set_ = 1.20 nA, and *V*_mod_ = 71 μV_rms_. Scale bar, 10 nm. (c and d) Spatial evolution of the ring patterns as a function of energy, corresponding to the purple and green box in panels a and b, respectively. (e) −d^3^*I*/d*V*^3^ spectra taken along the blue arrow in panels a and b (each line averaged over 3 × 3 pixels in the map).

**Figure 3:**
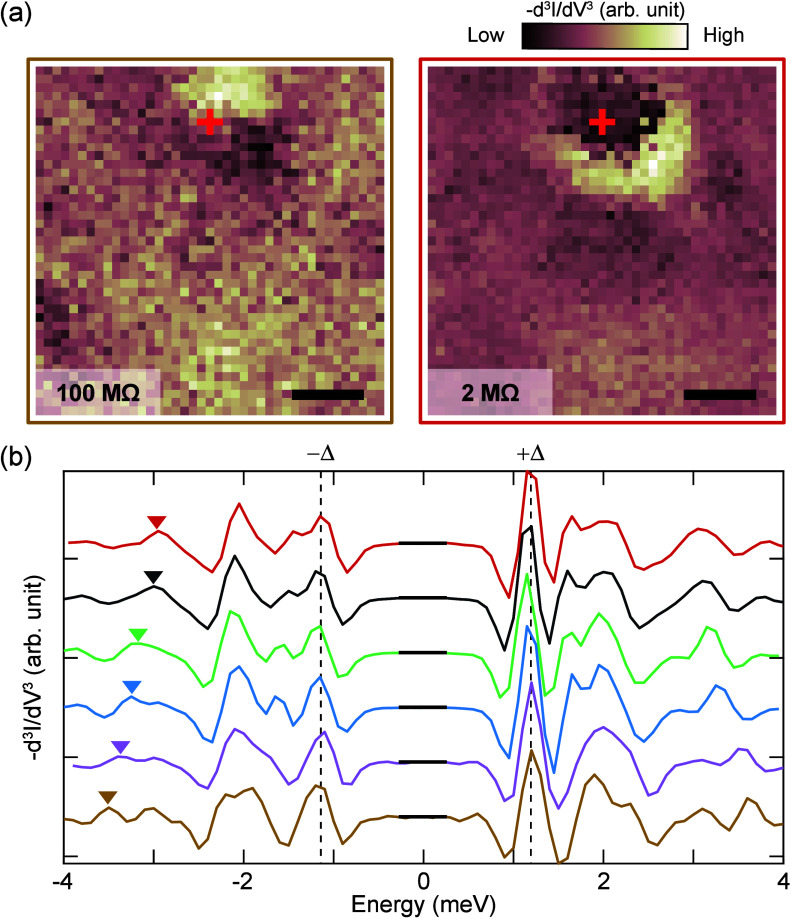
*R*_J_-dependent energy dispersion of the out-gap state. (a) *R*_J_-dependent spatial extent of the out-gap impurity state at the energy of +3.5 mV. Scale bar, 1 nm. (b) Point −d^3^*I*/d*V*^3^ spectra obtained at the location marked with a red cross in panel a with decreasing *R*_J_ [*R*_J_ = 2 (top), 2.5, 5, 10, 50, and 100 MΩ (bottom)]. Each curve is spatially averaged within 3 × 3 pixels and then shifted for clarity (zero values are indicated by black solid lines).

Figure 1c shows the topography of a cleaved surface with a square lattice composed of chalcogen atoms. The absence of protrusions in the STM image and the high *T*_c_ of our samples indicates that our sample includes very little excess Fe atoms^3,25,26^ – which suppresses *T*_c_(23) – consistent with previous STM studies.^12−14^Figure 1d shows a few spectra acquired at the sites marked with colored dots in Figure 1c in agreement with previous work.^3,13,14^ While the average spectrum (black curve in Figure 1d) looks somewhat similar to the Bogoliubov DOS expected from a single-gap Bardeen–Cooper–Schrieffer superconductor, our high-energy-resolution spectroscopy reveals that there is more than one peak in most single spectra (colored curves in Figure 1d). As the number of peaks and their energy change with location, the average spectrum will appear as if there is a single coherence peak with a broad hump (or shoulder). While these spectral features have previously been attributed to multiple superconducting gaps at different bands,^13^ we show here that our tunneling spectra are neither a reflection of a single gap nor of a series of coherence peaks. Instead, we argue that these spectral peaks stem from generalized Shiba states.

The first argument against the interpretation that the peaks manifest multiple coherence peaks at different bands stems from the ring-like features (Figure 2a) for the negative second derivative of the d*I*/d*V* spectra (−d^3^*I*/d*V*^3^; see Figures S4 and S5 for the same plots with d*I*/d*V* spectra. This is reminiscent of the generalized Shiba states observed previously at much lower energies of 0.8 meV (inside the lowest energy peak) and at much lower densities.^15^ There, similar ring-like features can be explained by impurity levels influenced by tip-gating. Looking at the peaks outside Δ_1_, we observe similar ring-like features (Figure 2b). They are overlapping due to their high density. Panels c–e of Figure 2 reveal that the ring-like features are attributed to spatially dispersive spectral features (Figure S6) with particle–hole symmetry (Figure S7); that is, their sizes vary depending on energy. Each impurity manifests different dispersion (Figure S6), which we attribute to different screenings of the tip-gating due to inherent inhomogeneity in carrier density. While the observed particle–hole symmetry further suggests their association with a superconducting state, their spatially dispersive behaviors contradict expectations of superconducting coherence peaks. Instead, they point to similar physics as seen in previously reported in-gap Shiba states,^15^ while originating from a much denser set of impurities. We note the particle–hole asymmetry in the Shiba state intensities (Figure 3a), which, in general, can be attributed to the broken particle–hole symmetry in the scattering potential of the impurity in superconductors.^27^ However, here we attribute this to particle-hole asymmetry in the DOS in the normal state of FeTe_0.55_Se_0.45_,^3^ which results in overall asymmetric spectral intensity, also observed in the coherence peak intensities. We further note that the absence of the spatial correlation between in-gap and out-gap Shiba states indicates that they have different origins.

The second argument stems from the junction resistance (*R*_J_) dependence. We compare the −d^3^*I*/d*V*^3^ maps at the energy of +3.5 meV obtained with vastly different *R*_J_ in Figure 3a. We obtain all these data with constant-current mode to sustain the same *R*_J_ for long-term measurement while keeping the same set bias (*V*_set_) and increasing (decreasing) the set current (*I*_set_) to decrease (increase) the *R*_J_. It is noticeable that the ring-like patterns change depending on the *R*_J_; it becomes larger (smaller) when the *R*_J_ is smaller (larger). This is because the energy level of the impurity state is tuned by the *R*_J_, as shown in Figure 3b. If the tip is brought closer to the sample (lower *R*_J_), a larger electric field is applied at the surface with the same *V*_set_. This is not surprising because the potential caused by the electric field from the STM tip is expected to be a function of the distance between the tip and the impurity, and it can vary as we move the tip either in a lateral or vertical direction.^15,28^ Thus, we have a similar trend in the size of the ring-like feature when we look at different energies with the fixed *R*_J_ and when we look at different *R*_J_ with the fixed *V*_set_. We emphasize that while the ring-like features and their dispersions are not the intrinsic properties of our system—since they are the consequence of the tip gate—these features allow us to distinguish the impurity states from coherence peaks. Further, the size of ring-like features yield the spatial extent of the impurity states that are independent of the presence of the tip.

The third argument stems from the spectral shapes and their spatial variations. In the absence of impurities, we would expect that each gap decreased the DOS further, leading to the highest intensity of the outermost peak, as observed in the multiband superconductors LiFeAs^10^ or NbSe_2_.^11^ However, this is not the case for most (∼90%) of our spectra showing the highest intensity at the smallest superconducting gap. This could be explained by tunneling matrix elements determined by constituent orbitals of the multibands.^29−31^ This scenario would, however, require that the large gap is on the d_*xy*_ orbital, which is in-plane and exhibits smaller tunneling,^32^ an unexpected situation for iron-based superconductors where the large gap is usually on the d_*xz*_/d_*yz*_ orbitals. Additionally, in the strained surface of LiFeAs, the d*I*/d*V* spectra also exhibit enhanced spectral weight at the smallest superconducting gap and suppression of the outermost peak.^10^ This suggests a possible link between the inhomogeneity caused by local strain from different configurations of chalcogen atoms^14^ and the observed suppression of the outermost peak, which we discuss below. Moreover, the fact that the number of peaks varies across locations disagrees with the expectation from a multiband superconductor.

Taken together, our data is inconsistent with the hypothesis of the extra peaks in the DOS of FeTe_0.55_Se_0.45_ being the coherence peaks of a multiband superconductor. We further mention the inconsistency with another possibility: inelastic tunneling due to bosonic modes, which could lead to the appearance of extra peaks. In such cases, the additional peaks follow the sharp coherence peak with a certain energy spacing corresponding to the energy scale of the bosonic modes, e.g., phonons^33,34^ or spin fluctuations,^35,36^ involved in pairing electrons. However, except for a few positions, there is no fixed energy between the peaks. Furthermore, such peaks from inelastic tunneling would likely be visible above the critical temperature as well,^37^ in disagreement with previous STM measurements on FeTe_0.55_Se_0.45_.^3^

Instead, we argue here that the phenomenology in FeTe_0.55_Se_0.45_ points toward an amorphous Shiba state causing a spectral weight transfer from the coherence peak of the larger superconducting gap to a set of Shiba states within this gap but larger than the smallest superconducting gap. These states overlap—we discuss the possibly important consequences of this fact at the end of our Letter. To consider such a scenario with the peaks being generalized Shiba states, we now have to address (i) why the coherence peak of the larger gap is not visible, (ii) how impurity-bound states can exist outside of the smallest superconducting gap (Δ_1_), and (iii) whether realistic parameters for impurity states can lead to extended peaks at energies *E* > Δ_1_. To answer these questions, we turn to theoretical simulations.

For our simulation of the strongly disordered FeTe_0.55_Se_0.45_ system, we use a generic five-band model with three orbitals, d_*xy*_, d_*xz*_, and d_*yz*_, present at low energies. Employing an interorbital and intraorbital order parameter with tetragonal symmetry, two energy scales are associated with superconductivity. In the order parameter of s_±_ symmetry at least two gap scales Δ_1_ and Δ_2_ occur, while the three-gap structure has been suggested by photoemission experiments.^9^ Despite discrepancies in the details, our model remains valid to describe the redistribution of spectral weight in the disordered system. We treat the impurities in the single-impurity approximation and include two different types of nonmagnetic scatterers. Note that magnetic scatters would lead to a similar phenomenology. To this end, we set up a *T*-matrix calculation of the LDOS where the local impurity potential is influenced by the presence of the STM tip by the induced gating potential *V*_imp_(**r**), where **r** is the distance of the STM tip to the impurity. The DOS picked up by the STM tip is calculated for a single impurity, and the density modulations are superimposed for a representative distribution of impurities (see the Supporting Information for details). The homogeneous DOS features two coherence peaks close to Δ_1_ and Δ_2_ (Figure 4a), with the larger gap removing around half of the spectral weight. This is reminiscent of clean multiband superconductors such as LiFeAs,^10^ but very different from our observations in FeTe_0.55_Se_0.45_. Instead, as discussed below, the strongly disordered network of overlapping Shiba states (Figure S8) alters the DOS to a single-gap DOS with only one visible peak at Δ_1_.

**Figure 4:**
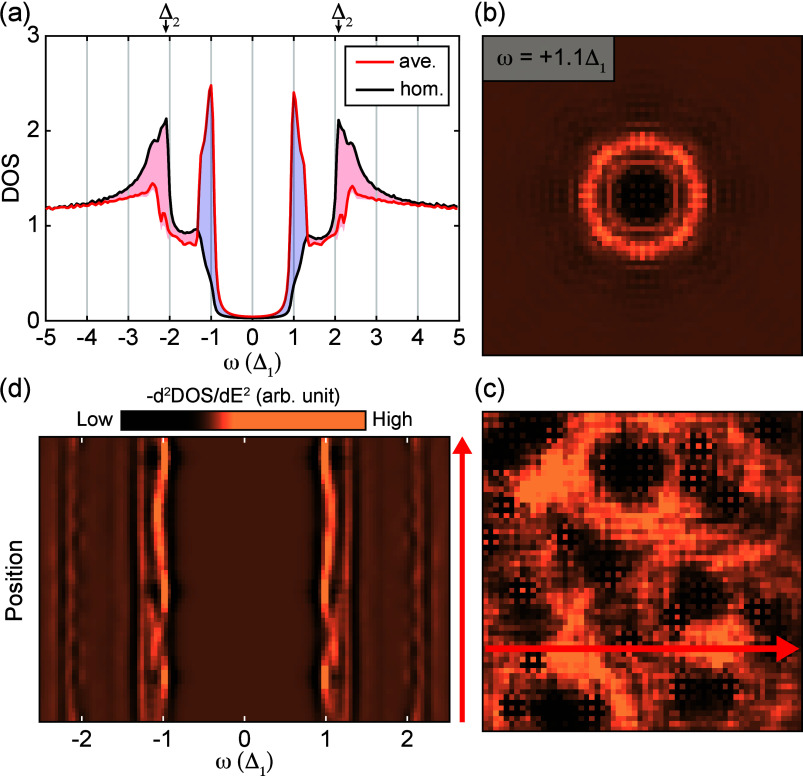
Orbital-selective pair-breaking in a multiband Fe-based superconductor. (a) DOS of the homogeneous system compared to the average DOS of the inhomogeneous system where the coherence peak at Δ_1_ is enhanced (blue shaded), while the coherence peak at Δ_2_ (≈2.08Δ_1_) is suppressed (red shaded). (b and c) Negative second derivative of LDOS maps on a field of view of 60 × 60 lattice constants at the energy ω = 1.1Δ_1_, showing a single impurity and 30 impurities, respectively. (d) Negative second derivative of the LDOS along the cut marked by the red arrow in panel c, showing inhomogeneous peaks and the spatial dispersion from the generalized Shiba states at ω > Δ_1_ (each line averaged over 3 × 3 pixels in the map).

Figure 4 summarizes the outcome of the theoretical simulations of the strongly disordered FeTe_0.55_Se_0.45_ superconductor. Figure 4a compares the spatially averaged DOS, whereas panels b and c of Figure 4 display the LDOS maps for a single impurity and multiple ones at energies between Δ_1_ and Δ_2_. Finally, in Figure 4d, we show representative cross sections. Additional cross-sections of other impurity configurations are provided in the Supporting Information, where we also discuss the properties of single impurities of two different types.

The simulations for multiple impurities display a striking resemblance to our data in several key aspects. First, the nonmagnetic impurities produce in-gap states, as also observed experimentally. Second, due to the gating effect of the STM tip, the states disperse as a function of tip position and therefore extend as rings of several nanometers around the impurities if examined as conductance maps, see Figure 4b. Third, and perhaps most surprisingly, the superposition of impurity-bound states (Figure 4c) and associated peaks and dips in the spectrum leads to a strong modification of the homogeneous DOS as shown in Figure 4a. The spectral weight of the coherence peak at Δ_2_ is drained (red shaded area), leaving only a faint trace; while the peak close to Δ_1_ is strongly enhanced (blue shaded area) rendering it similar in overall appearance to the spectrum of a single-gap superconductor, at least when viewed from the spatially averaged spectrum (Figure 1d). All these features are seen in the experimental data as well. For a more detailed comparison, we also provide spectra along cuts in real space where it is visible that the impurity-bound states of the strong impurities disperse at energies below Δ_1_ (see the Supporting Information), while there are dispersing peaks beyond the energy scale Δ_1_ stemming from the weak type of impurities.

The scattering from the potential disorder occurs only between states on the Fermi surface where the orbital character is identical (Figure S9). To induce Shiba in-gap states, the scattering has to be additionally sign-changing (Figure S10); otherwise, the superconducting state is protected from in-gap states due to Anderson’s theorem. Note that our modeling uses a large impurity potential for one type of scatterer, diagonal in orbital space which could be associated with defects on the Fe site (also below the surface layer) and will therefore act as a strong scatterer. The other weak impurity is chosen to only affect the d_*xz*_ and d_*yz*_ orbitals associated with a larger gap in our model (Figure S10) because the orbitals can be coupled to impurities away from the Fe plane. This yields the phenomenology of not inducing any bound states at energies below Δ_1_ (as in the experimental data). This choice is compatible with the effects of the disorder at Se/Te sites. Disorder in the chalcogen layer mainly affects the interlayer coupling of the d_*xz*_ and d_*yz*_ orbitals since these hybridize with the Se/Te atoms out of the plane. In contrast, the d_*xy*_ orbital of Fe couples mostly to the neighboring Fe atoms and less in the out-of-plane direction. Thus, we propose that the scattering is orbital-selective, dominantly couples to the states on the Fermi surface where the order parameter is large, i.e., beyond the scale Δ_1_, and therefore effectively suppresses signatures from the larger order parameter Δ_2_. This picture indicates that the disorder created by the FeSe/FeTe alloying could play a role. To simulate this, one would need a full real-space calculation in the field of view, which is out of reach with our current calculation power. At this point, we are agnostic to the origin of the scatterers, and note that Te/Se disorder or nonperiodic spin structures on the iron atoms could play a role. The latter is reminiscent of experiments with magnetic islands on a conventional superconductor Nb(110) surface.^38^ Taken together, the similarity between the simulations and data in all aspects is strong.

To conclude our Letter, we discuss the ramifications of a set of Shiba states to the physics of FeTe_0.55_Se_0.45_. The key aspect is that the states we detect are randomly distributed and have a strong spatial overlap. If these states are hybridized, this would lead to an amorphous Shiba band, not in the sense of a Bloch band but similar to the impurity band in semiconductors. It would be without periodicity, embedded in a crystal lattice, and coexisting with electronic Bloch bands from said lattice. The interconnected impurities result in an unusual Hamiltonian, potentially leading to topologically nontrivial states in amorphous matter.^39−41^ We do observe a few cases where two isolated but nearby Shiba states seem to be hybridized (Figure S8), but generally, the high density of Shiba states and their complexity in energy and position obscure such signatures. It would also be highly interesting to revisit this issue by engineering amorphous networks on low-dimensional metal surfaces by atomic manipulations^42−47^ and compare the results to FeTe_0.55_Se_0.45_. In any case, our work implies that the low-energy quasiparticle continuum of FeTe_0.55_Se_0.45_ is very distinct from that arising from a basic hopping-on-a-lattice model relevant to homogeneous multigap superconductors, and instead represents an intriguing new configuration of electron matter.

## Supporting Information Available

Details on STM/S measurements and theoretical calculations; raw and deconvoluted STS data; analysis of particle–hole symmetry in generalized Shiba states and signatures of their hybridization; orbital-decomposed band structure and superconducting order parameters; and theoretical exploration of various possible impurity configurations (PDF)
