# Supplementary material for: Signatures of Amorphous Shiba State in FeTe0.55Se0.45
Source: Nano Lett. 2025 Feb 12;25(11):4227–33. doi: 10.1021/acs.nanolett.4c05650 (PMC11926944; doi:10.1021/acs.nanolett.4c05650)
Supplement: Supplementary file 1 — nl4c05650_si_001.pdf [file nl4c05650_si_001.pdf]

Supporting Information for "Signatures of Amorphous Shiba State in $FeTe_{0.55}Se_{0.45}$"

Jinwon Lee,[†,@] Sanghun Lee,[‡,@] Andreas Kreisel,[¶,@] Jens Paaske,[¶] Brian M. Andersen,[¶] Koen M. Bastiaans,[†] Damianos Chatzopoulos,[†] Genda Gu,[§] Doohee Cho,[*,‡] and Milan Allan[*,†]

†Leiden Institute of Physics, Leiden University, Leiden, 2333 CA, The Netherlands

‡Department of Physics, Yonsei University, Seoul, 03722, South Korea

¶Niels Bohr Institute, University of Copenhagen, DK-2200 Copenhagen, Denmark

§Condensed Matter Physics and Materials Science Department, Brookhaven National Laboratory, Upton, NY 11973, USA

‖Faculty of Physics, Ludwig-Maximilians-University Munich, Munich 80799, Germany

⊥Center for Nano Science (CeNS), Ludwig-Maximilians-University Munich, Munich 80799, Germany

#Munich Center for Quantum Science and Technology (MCQST), Ludwig-Maximilians-University Munich, Munich 80799, Germany

@These authors contributed equally to this work.

E-mail: dooheecho@yonsei.ac.kr; allan@physics.leidenuniv.nl

Supplementary Note 1: STM measurement

$FeTe_{0.55}Se_{0.45}$ single crystals ($T_c \approx 14.5$ K) are grown by using the Bridgman method. For STM and STS measurements, we employ a modified commercial scanning tunneling microscope (USM-1500, Unisoku Co., Ltd). The samples are cleaved in an ultrahigh vacuum ($P \approx 1 \times 10^{-10}$ mbar) at low temperature ($T \approx 30$ K) and are quickly transferred to the pre-cooled STM head ($T \approx 2.2$ K) to avoid surface reconstruction and contamination. Mechanically sharpened Pt–Ir wires are used for STM tips. To make a superconducting tip, the metallic tips are indented into a Pb(111) surface until the tunneling spectrum shows the U-shaped gap with the sharper coherence peak at the energy of $\Delta_{\rm tip} + \Delta_{\rm sample} = 2.66$ meV. To ensure the stability of the superconducting tip, we conducted STS measurements, gradually lowering the junction resistance until it reached approximately 0.14 MΩ. For the tunneling spectrum measurements, we used a standard lock-in technique, and all the necessary parameters for the measurements are provided in the figure captions. We use the set bias larger than the superconducting gap to minimize changes in the tip height due to heterogeneous impurity states.

Supplementary Note 2: Homogeneous superconductor

In order to describe the amorphous impurity band from a microscopic theoretical model, we start with an electronic structure relevant for $FeSe_{1-x}Te_x$ which is a five-orbital model given by the tight-binding Hamiltonian

$$\mathcal{H}_0 = \sum_{\substack{\mathbf{k}\sigma \\ \alpha\beta}} h_{\alpha\beta}(\mathbf{k}) c^\dagger_{\mathbf{k}\sigma\alpha} c_{\mathbf{k}\sigma\beta} \,, \tag{1}$$

where $c_{\mathbf{k}\sigma\beta}$ is the Fourier amplitude of an operator $c_{i\sigma\beta}$ that creates an electron in Wannier orbital α with spin σ, and $h_{\alpha\beta}(\mathbf{k})$ is the Fourier transform of the hopping elements (including the chemical potential μ) connecting states α and β.[1] The band structure in fat band scheme and the Fermi surface are presented in Figure S9. In the same figure, we illustrate the differences between the representation of the normal state electronic structure in a setting

with 1 Fe atom per elementary cell (b) and the setting with 2 Fe per elementary cell (c). The latter is the electronic structure that would be measured experimentally, for example in an ARPES experiment. For the theoretical calculations we use the 1 Fe setting which has an exact mapping to the 2 Fe setting at least in calculations of two dimensional models, a good approximation for the layered Fe-based materials. This model, originally derived for LiFeAs has been examined for superconducting instabilities within a self-consistent Bogoliubov-de-Gennes (BdG) approach to obtain the superconducting order parameter $\Delta_{\alpha\beta}(\mathbf{k})$ in orbital space. In this work, we simply adopt this order parameter that is presented in Figure S10 together with the tight-binding model to obtain the BdG Hamiltonian

$$\mathcal{H}_{\mathrm{BdG}} = \sum_{\substack{\mathbf{k} \\ \alpha\beta}} \Psi^{\dagger}_{\mathbf{k}\alpha} H(\mathbf{k})_{\alpha\beta} \Psi_{\mathbf{k}\beta} \,, \tag{2}$$

where $\Psi^{\dagger}_{\mathbf{k}\alpha} = \begin{pmatrix} c^{\dagger}_{\mathbf{k}\uparrow\alpha} & c_{-\mathbf{k}\downarrow\alpha} \end{pmatrix}$ and the BdG matrix given by

$$H(\mathbf{k})_{\alpha\beta} = \begin{pmatrix} h_{\alpha\beta}(\mathbf{k}) & -\Delta_{\alpha\beta}(\mathbf{k}) \\ -\left[\Delta_{\alpha\beta}(\mathbf{k})\right]^{\dagger} & -h_{\beta\alpha}(-\mathbf{k}) \end{pmatrix} . \tag{3}$$

It describes a homogeneous superconductor including relevant multiorbital-multiband physics together with an order parameter of sign-changing s-wave type that exhibits the two scales scales of the order parameter. $\Delta_1 = 6\,\mathrm{meV}$ marks the minimal gap on the Fermi surface and $\Delta_2 = 12.5\,\mathrm{meV}$ marks the largest coherence peak in the spectrum, see Figure S10. Indeed, the gap structure has further maxima on the various Fermi surface sheets which lead to additional (small) van Hove singularities, i.e. peaks in the density of states. The conclusions from our theoretical calculations are, however, expected to not depend on additional gap scales as long as the model exhibits two gap scales which originate from two different orbital channels such that multiple in-gap bound states can form. Note that the band structure is unrenormalized, i.e. Fermi velocities and order parameter magnitudes are nominally

larger than in the real material. In the following, we use Δ_1 as the relevant energy scale for comparing to experimental data.

Supplementary Note 3: T-matrix approach

To calculate the density of states in the presence of a single impurity, we use the T-matrix approach where the Green function in real-space is given by

$$G(\mathbf{r},\mathbf{r}',\omega) = G^{(0)}(\mathbf{r}-\mathbf{r}',\omega) + G^{(0)}(\mathbf{r})T(\mathbf{0},\omega)G^{(0)}(-\mathbf{r}',\omega) \tag{4}$$

with the T-matrix defined by

$$T_i(\mathbf{0},\omega) \equiv (1 - H_{\mathrm{imp},i}G^{(0)}(\mathbf{0},\omega))^{-1}H_{\mathrm{imp},i} \tag{5}$$

where the matrix $H_{\mathrm{imp},i}$ describes a point-like impurity of type i. The Green function in real space is calculated by a Fourier transform $G^{(0)}(\mathbf{r},\omega) = \sum_{\mathbf{k}} G^{(0)}(\mathbf{k},\omega)e^{i\mathbf{k}\cdot\mathbf{r}}$ from the retarded Green function $G^{(0)}(\mathbf{k},\omega) = [\omega + i\eta - H(\mathbf{k})]^{-1}$. We can then calculate the electronic LDOS at position $\mathbf{r}$ (of one spin species) by calculating the partial trace

$$\rho(\mathbf{r},\omega) = -\frac{1}{\pi}\sum_{\alpha}\mathrm{Im}(G_{\alpha\alpha}(\mathbf{r},\omega))\,. \tag{6}$$

The density modulations from one single impurity are then given by

$$\delta\rho_i(\mathbf{r},\omega) = -\frac{1}{\pi}\sum_{\alpha}\mathrm{Im}(G^{(0)}(\mathbf{r})T_i(\mathbf{0},\omega)G^{(0)}(-\mathbf{r},\omega))\,. \tag{7}$$

Supplementary Note 4: Gating and impurity potential

In our approach, the impurity potential is modified by the presence of the STM tip in the sense that the value of the impurity potential is modified as the tip approaches both in

z-direction and within the x-y plane. We assume a behavior of the following form

$$V_\alpha(\mathbf{r}) = V_{\alpha,0} + \frac{V_{\alpha,g}}{\sqrt{\mathbf{r}^2/r_0^2+1}}, \tag{8}$$

where $V_{\alpha,0}$ is the potential in the absence of the STM tip and $V_{\alpha,g}$ is the effective charge of the STM tip which is located at a height r_0 and at the position $\mathbf{r}$. Guided by the experimental finding that two qualitatively distinct types of impurity states are detected, we incorporate also two different types of scatterers in the modelling. The first defect type induces an in-gap state and a clearly visible ring-shape in the conductance maps (if the STM tip is close enough). For this impurity, we use

$$H_{\mathrm{imp},1} = \tau_z \otimes \begin{pmatrix} V_1 & 0 & 0 & 0 & 0 \\ 0 & V_1 & 0 & 0 & 0 \\ 0 & 0 & V_1 & 0 & 0 \\ 0 & 0 & 0 & V_1 & 0 \\ 0 & 0 & 0 & 0 & V_1 \end{pmatrix} \tag{9}$$

with a large potential $V_{1,0} = 0.25$ eV, $V_{1,g} = 1.1$ eV and $r_0 = 10\,a$. We measure all distances in units of the NN Fe-Fe bond length that also plays the role of the lattice constant in our five-band model. The other type of impurity appears weaker and only induces visible modulations of the density of states above the gap scale of Δ_1. Therefore, we assume the impurity potential to be only on the d_{xz} and d_{yz} orbital such that no bound state is created in the d_{xy} channel. Such an effective impurity potential is expected to arise from defects away from the Fe plane, i.e. related to the Se/Te disorder. This disorder is located above the centers of the squares formed by the Fe atoms. Observing that the NNN hoppings across the center of these squares is very small for the d_{xy} orbital of ~ 10 meV while the NNN hoppings of the d_{xz} and d_{yz} orbitals reach ~ 250 meV, it is expected that such disorder interrupts the hoppings of the mentioned orbitals. We effectively take this into account by an orbital

dependent scatterer with the matrix structure

$$H_{\mathrm{imp},2} = \tau_z \otimes \begin{pmatrix} 0 & 0 & 0 & 0 & 0 \\ 0 & 0 & 0 & 0 & 0 \\ 0 & 0 & V_2 & 0 & 0 \\ 0 & 0 & 0 & V_2 & 0 \\ 0 & 0 & 0 & 0 & 0 \end{pmatrix} . \tag{10}$$

We choose a smaller potential $V_{2,0} = 0.3$ eV, $V_{2,g} = 0.6$ eV, and a larger length scale $r_0 = 20\,a$ suggesting an impurity further down below the surface. In Figure S11 we show that the averaged spectra exhibit large peaks close to Δ_1 for a variation of these parameters. We note at this point that the putative "homogeneous spectrum" cannot be observed experimentally because of the strong disorder and the observation of the spectra with only one strong peak close to Δ_1 should not be interpreted as the scale of the order parameter being Δ_1. This is in line with the observation of the peak position 1.4 meV which is too small for a 14.5 K superconductor.

In Figures. S12 and S13, we show the LDOS and spectra of a single impurity of type 1 (strong) and type 2 (weak). The strong impurity exhibits dispersive in-gap states and a clearly visible ring-like structure in the LDOS, while the weak impurity displays modulations above the energy scale Δ_1 and a very faint ring-like feature at this energy scale as well. In Figure S14 we show variations of the in-gap Shiba states that reveal the dependence on the strength of the gating potential and tuneability of the Shiba state as well as the zero crossing, panel c which was earlier discussed in Ref.[2]

Supplementary Note 5: Modelling the amorphous impurity band

Ignoring the interference between different impurities, we calculate the total modulation

of the density of states by performing the following summation

$$\delta\rho(\mathbf{r},\omega)_{\mathrm{multi}} = \sum_{n} \delta\rho_1(\mathbf{r}-\mathbf{r}_{1,n},\omega) + \sum_{m} \delta\rho_2(\mathbf{r}-\mathbf{r}_{2,m},\omega) \tag{11}$$

where we sum over the n impurities of type 1 and the m impurities of type 2 which are randomly placed in the field of view. In Figure S15, we show more examples of averaged spectra (over the field of view), as well as spectra along cuts in real space and LDOS maps at two representative energies for two different impurity configurations.

References

(1) Kreisel, A.; Nelson, R.; Berlijn, T.; Ku, W.; Aluru, R.; Chi, S.; Zhou, H.; Singh, U. R.; Wahl, P.; Liang, R.; Hardy, W. N.; Bonn, D. A.; Hirschfeld, P. J.; Andersen, B. M. Towards a quantitative description of tunneling conductance of superconductors: Application to LiFeAs. *Phys. Rev. B* **2016**, *94*, 224518.

(2) Chatzopoulos, D.; Cho, D.; Bastiaans, K. M.; Steffensen, G. O.; Bouwmeester, D.; Akbari, A.; Gu, G.; Paaske, J.; Andersen, B. M.; Allan, M. P. Spatially dispersing Yu-Shiba-Rusinov states in the unconventional superconductor $FeTe_0.55Se_0.45$. *Nat. Commun.* **2021**, *12*, 1–8.

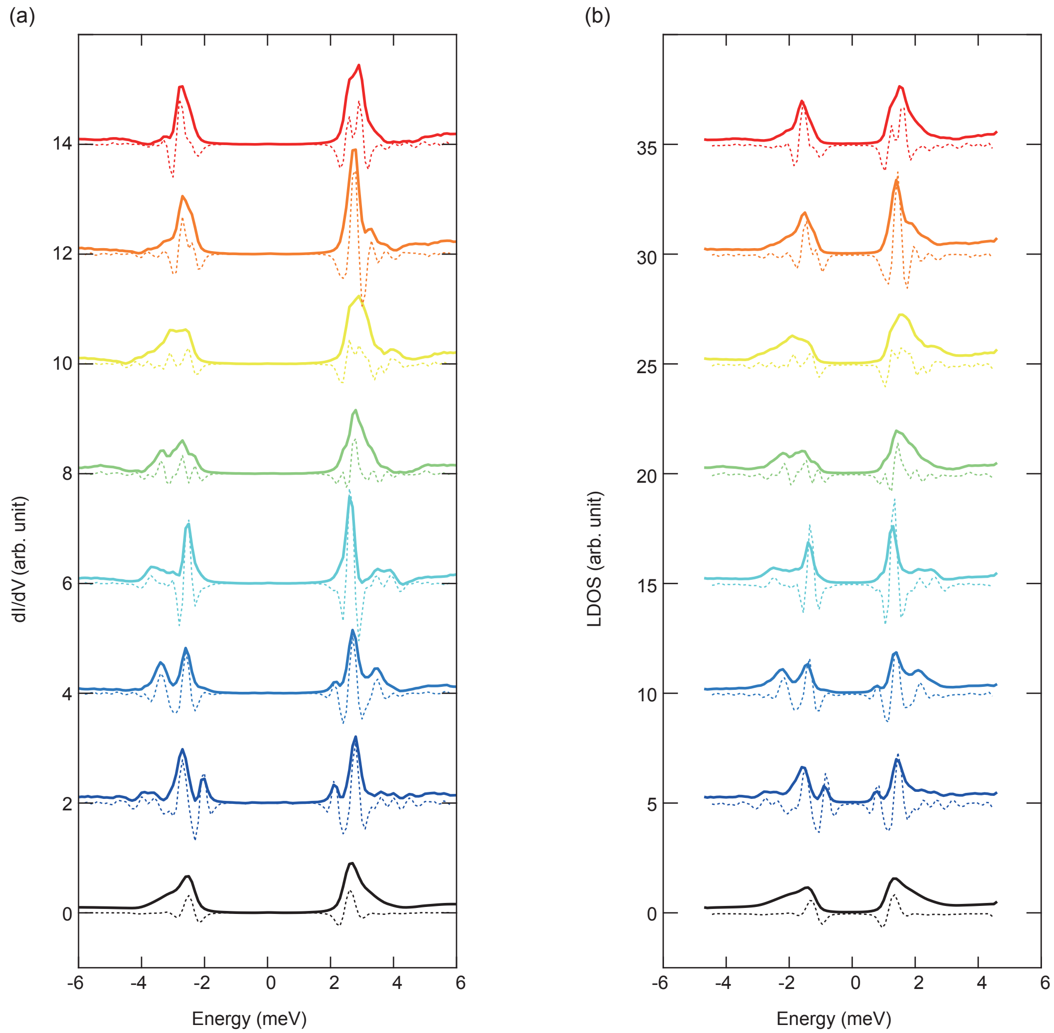

Figure S1: Comparison between measured and deconvoluted tunneling spectra and their second derivatives. (a) Tunneling spectra (solid curves) obtained with a Pb-coated STM tip at the positions marked by color circles in Figure 1(d). The dashed curves are the negative of second derivatives of the measured spectra ($-d^3I_m/dV^3$). (b) DOS spectra acquired by deconvoluting the tunneling spectra with the DOS of a superconducting tip. They are shown in Figure 1(e). The dashed curves are the negative of second derivatives of the DOS spectra ($-d^3I/dV^3$). Compared to the measured ones, all the spectral peaks in (b) are shifted by 1.3 meV corresponding to the pair-breaking energy of the Pb-coated tip. In the main text, we used the negative of second derivatives of the DOS spectra ($-d^3I/dV^3$) to emphasize the spectral peaks and their spatial variations, unless there is a specific statement.

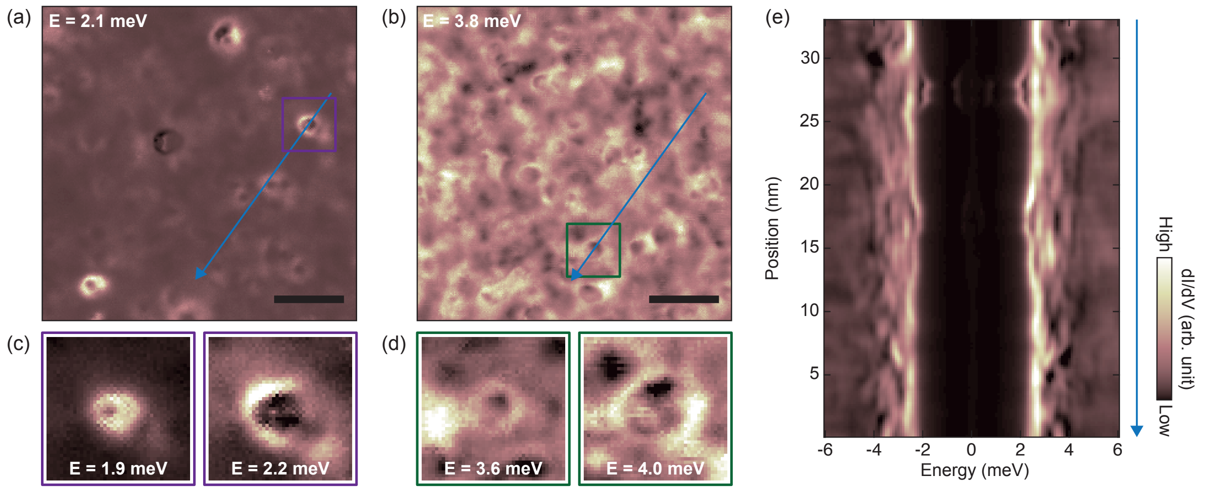

Figure S2: Spatially dispersive generalized Shiba states inside and outside the superconducting gap (raw data of Figure 2 and Figure S4). (a) and (b) Differential conductance (dI/dV) maps at the energies of $+2.1$ and $+3.8$ meV for the same field of view. The dI/dV maps are acquired with $V_{\rm set}$=-6.0 mV, $I_{\rm set}$=1.20 nA, dV=$V_{\rm mod}$=100 μV. Scale bar, 10 nm. (c) and (d) Spatial evolution of the ring patterns as a function of energy, corresponding to the purple and green box in (a) and (b), respectively. (e) The dI/dV spectra taken along the blue arrow in (a) and (b) (each line averaged over 3×3 pixel in the map).

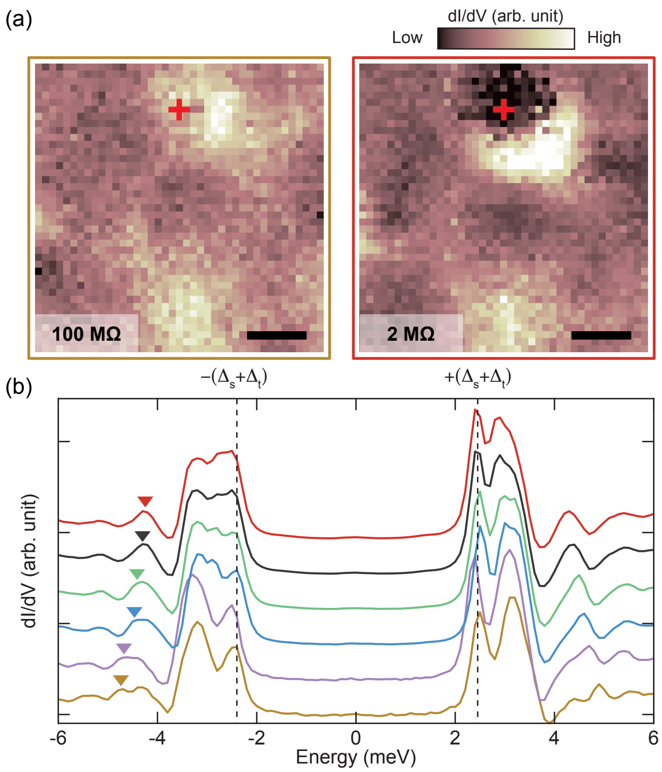

Figure S3: R_J-dependent energy dispersion of the out-gap state (raw data of Figure 3 and Figure S5). (a) R_J-dependent spatial extent of the out-gap impurity state at the energy of +4.8 meV. Scale bar, 1 nm. (b) Point dI/dV spectra obtained at the location marked with a red cross in (a) with decreasing junction resistances (R_J = 2 (top), 2.5, 5, 10, 50, and 100 MΩ (bottom)). Each curve is spatially averaged within 3 × 3 pixels, then shifted for clarity. By considering the particle-hole symmetry of the coherence peak, there is a shift lower than 100 μV due to the bias offset.

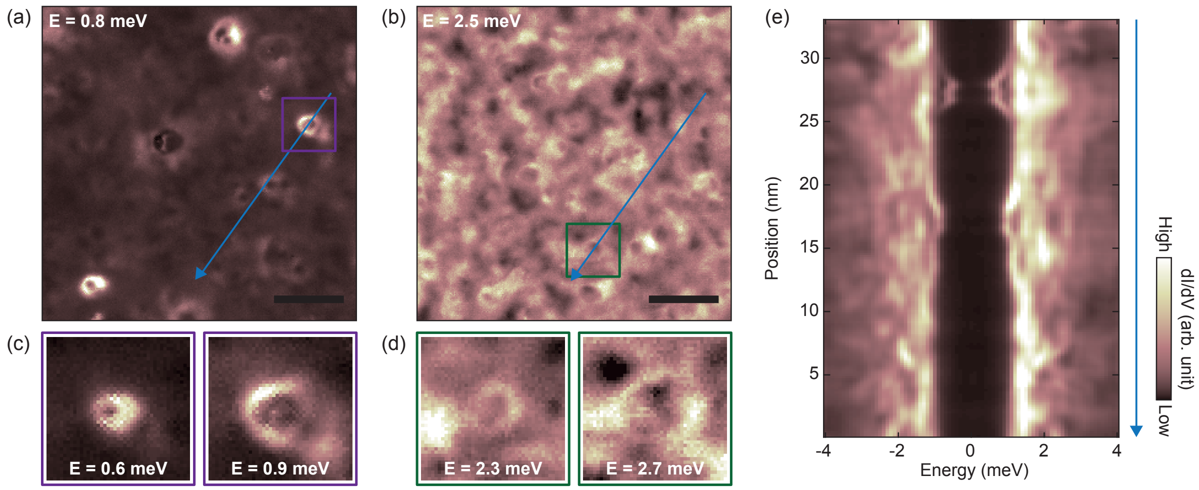

Figure S4: Spatially dispersive generalized Shiba states inside and outside the superconducting gap (deconvoluted data of Figure S2). (a) and (b) Differential conductance (dI/dV) maps at the energies of $+0.8$ and $+2.5$ meV for the same field of view. The dI/dV maps are acquired with $V_{\rm set}$=-6.0 mV, $I_{\rm set}$=1.20 nA, dV=$V_{\rm mod}$=100 μV. Scale bar, 10 nm. (c) and (d) Spatial evolution of the ring patterns as a function of energy, corresponding to the purple and green box in (a) and (b), respectively. (e) The dI/dV spectra taken along the blue arrow in (a) and (b) (each line averaged over 3×3 pixel in the map).

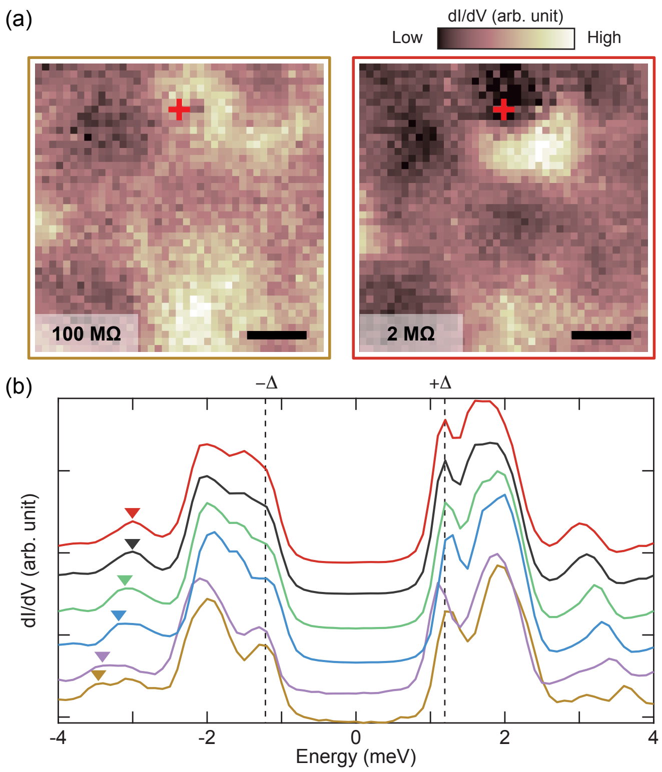

Figure S5: R_J-dependent energy dispersion of the out-gap state (deconvoluted data of Figure S3). (a) R_J-dependent spatial extent of the out-gap impurity state at the energy of +3.5 meV. Scale bar, 1 nm. (b) Point dI/dV spectra obtained at the location marked with a red cross in (a) with decreasing junction resistances (R_J = 2 (top), 2.5, 5, 10, 50, and 100 MΩ (bottom)). Each curve is spatially averaged within 3 × 3 pixels, then shifted for clarity. By considering the particle-hole symmetry of the coherence peak, there is a shift lower than 100 μV due to the bias offset.

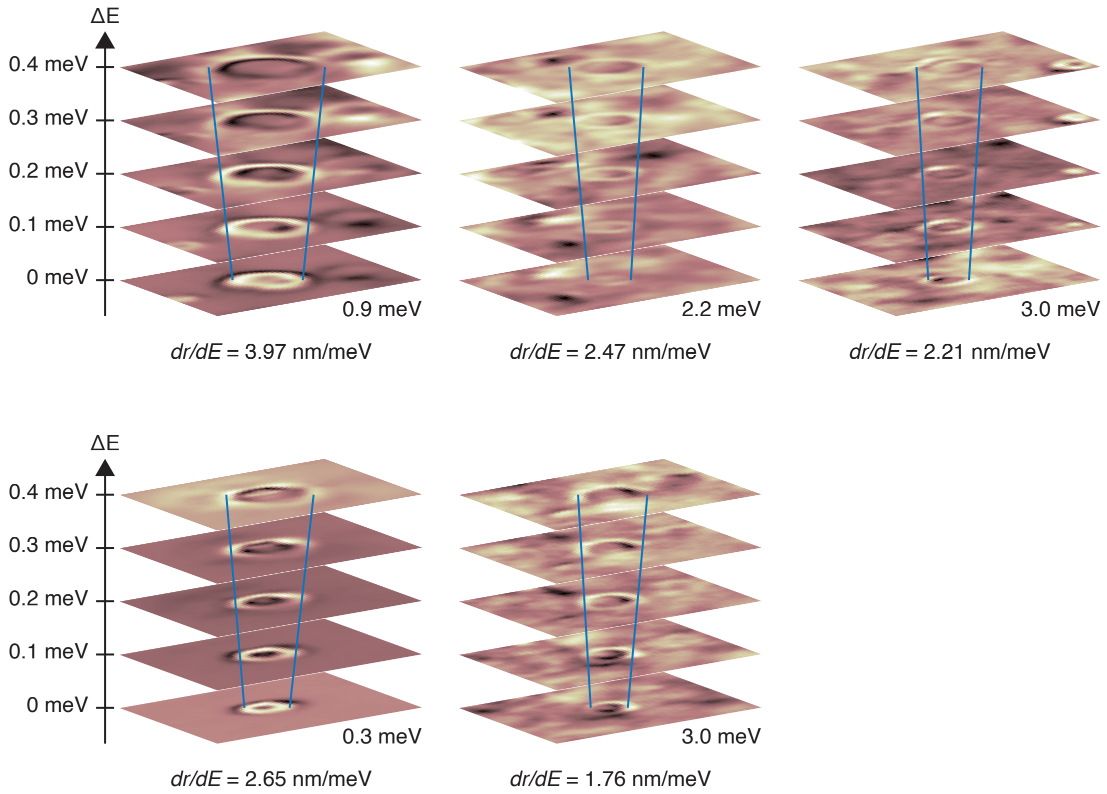

Figure S6: Energy-dependent dispersive behavior of generalized Shiba states inside and outside the first coherence peak.

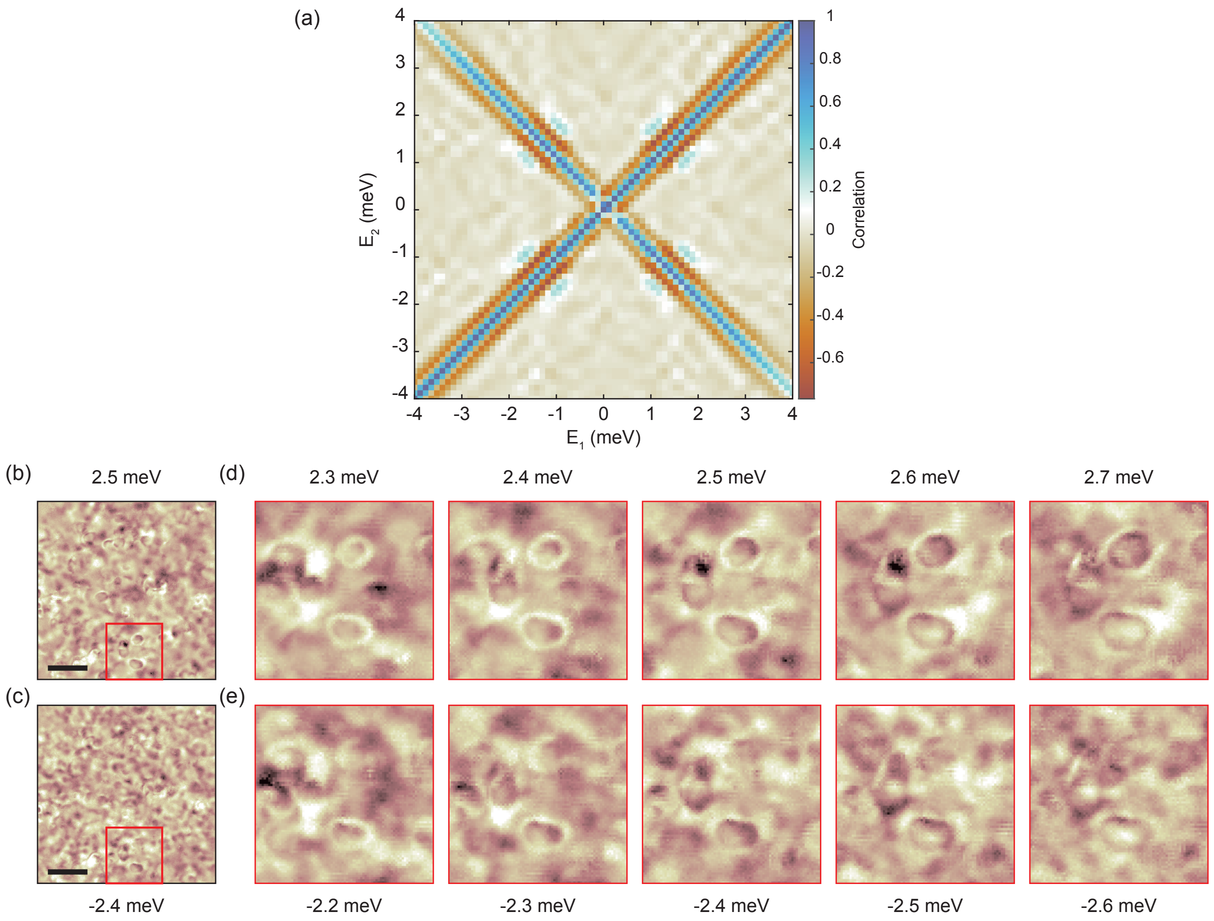

Figure S7: Particle-hole symmetry of the generalized Shiba states. (a) 2D correlation plot of a negative d^3I/dV^3 map. Each pixel represents the correlation coefficient between $-d^3I/dV^3(E = E_1)$ and $-d^3I/dV^3(E = E_2)$. (b) Real-space $-d^3I/dV^3$ map for energy layer of 2.5 meV. (c) $-d^3I/dV^3$ map at the same field of view as in (b) but with energy polarity reversed (-2.4 meV). (d(e)) Zoomed-in view of the red rectangle marked in (b(c)). Energy changes from 2.3 (-2.2) to 2.7 (-2.6) meV to demonstrate the $p - h$ symmetry in the spatial evolution of the ring-like features. Note that we compared energy layers differing by 0.1 meV due to a bias offset of 50 μV. (Scale bar, 10 nm)

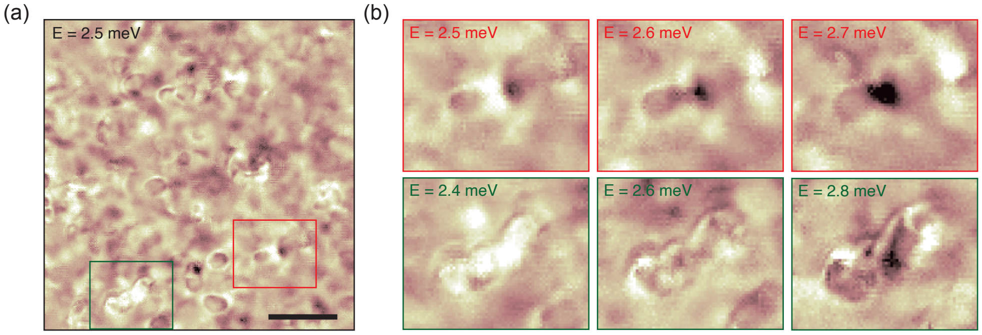

Figure S8: Hybridization of the impurity states. (a) A negative d^3I/dV^3 map shown in Figure 2 in the main manuscript. (b) Bias-dependent $-d^3I/dV^3$ maps for the area marked by colored box in (a). The ring-like features form arcs when they merge together. (Scale bar, 10 nm)

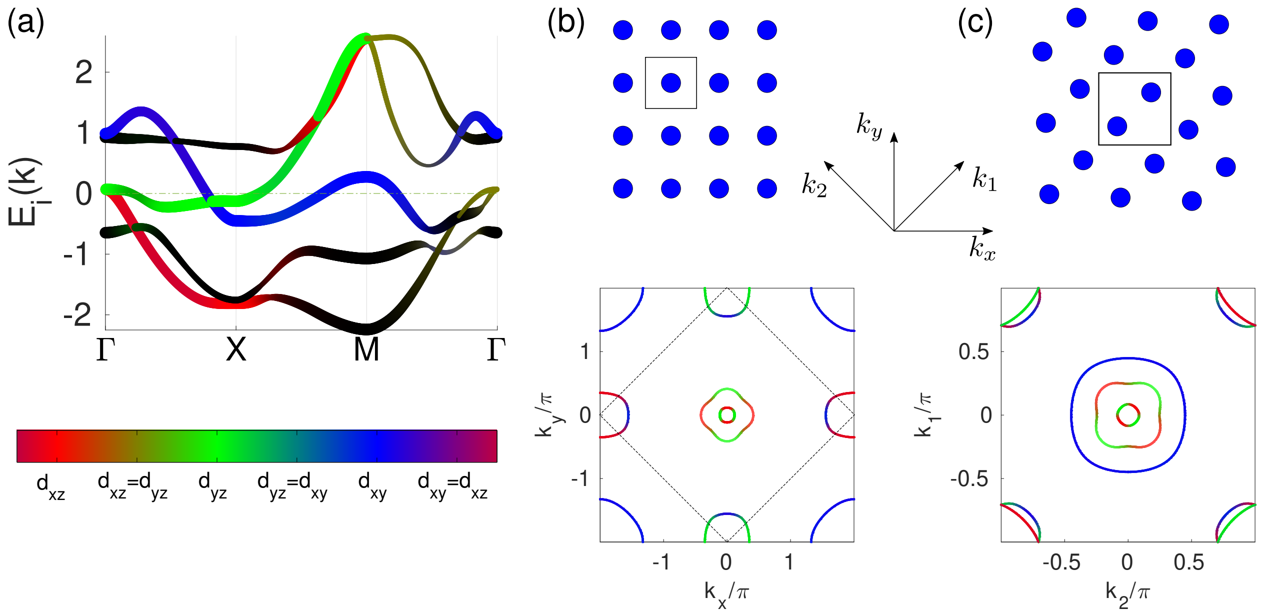

Figure S9: (a) Band structure of our tight binding model in a fat-band scheme with the orbital content of d_{xz}, d_{yz} and d_{xy} orbital indicated by colors. Comparison between 1Fe setting (b) and 2Fe setting (c): Lattice of Fe atoms (blue dots) together with elementary cell (black square). Fermi surface in the 1Fe setting where the boundary of the Brillouin zone of the 2 Fe setting is indicated as dashed lines (b, bottom panel). Fermi surface of the same model, but folded into the 2 Fe Brillouin zone with the rotated coordinate system (c, bottom panel).

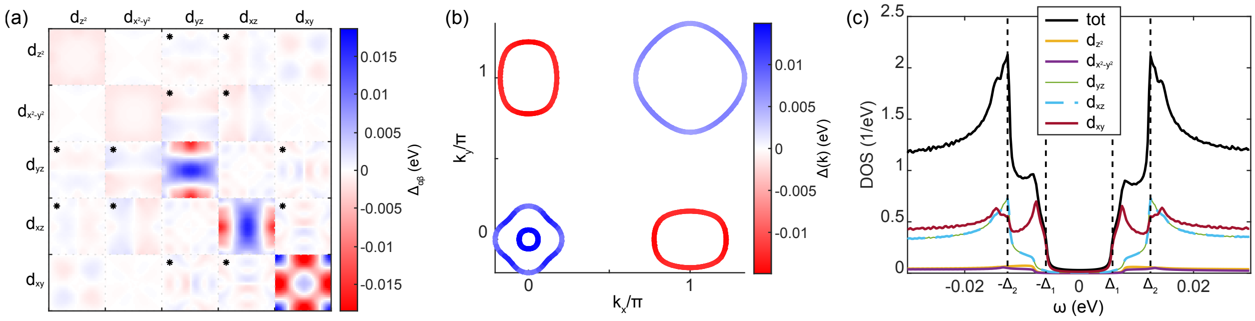

Figure S10: **Superconducting order parameter.** (a) The superconducting order parameter is obtained in orbital space from a microscopic calculation as in Ref.[1] (Order parameter for the k-points in the full Brillouin zone in each square, imaginary components marked with an asterisk "$*$".) (b) Transformation of order parameter into band space and prjoection on the Fermi surface reveals the sign-changing s-wave symmetry with large gap on the $d_{xz/yz}$ orbital and smaller gap on the d_{xy} orbital. (c) Density of states in the superconducting state of the homogeneous system with two expected coherence peaks, one slightly above Δ_1 and the other at Δ_2, the smaller gap magnitude predominantly on the d_{xy} orbital, the larger gap on the $d_{xz/yz}$ orbitals.

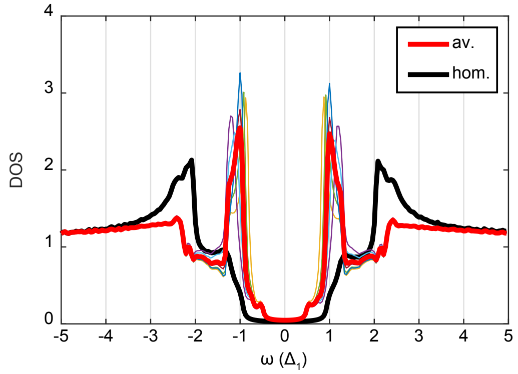

Figure S11: Averaged density of states of for different choices of weak impurities. Thick red line as in the main text and thin lines for $V_{2,0}$ increasing/decreasing by 20 %,$V_{2,g}$ increasing/decreasing by 20 % and increasing r_0 to $24a$ and $36a$.

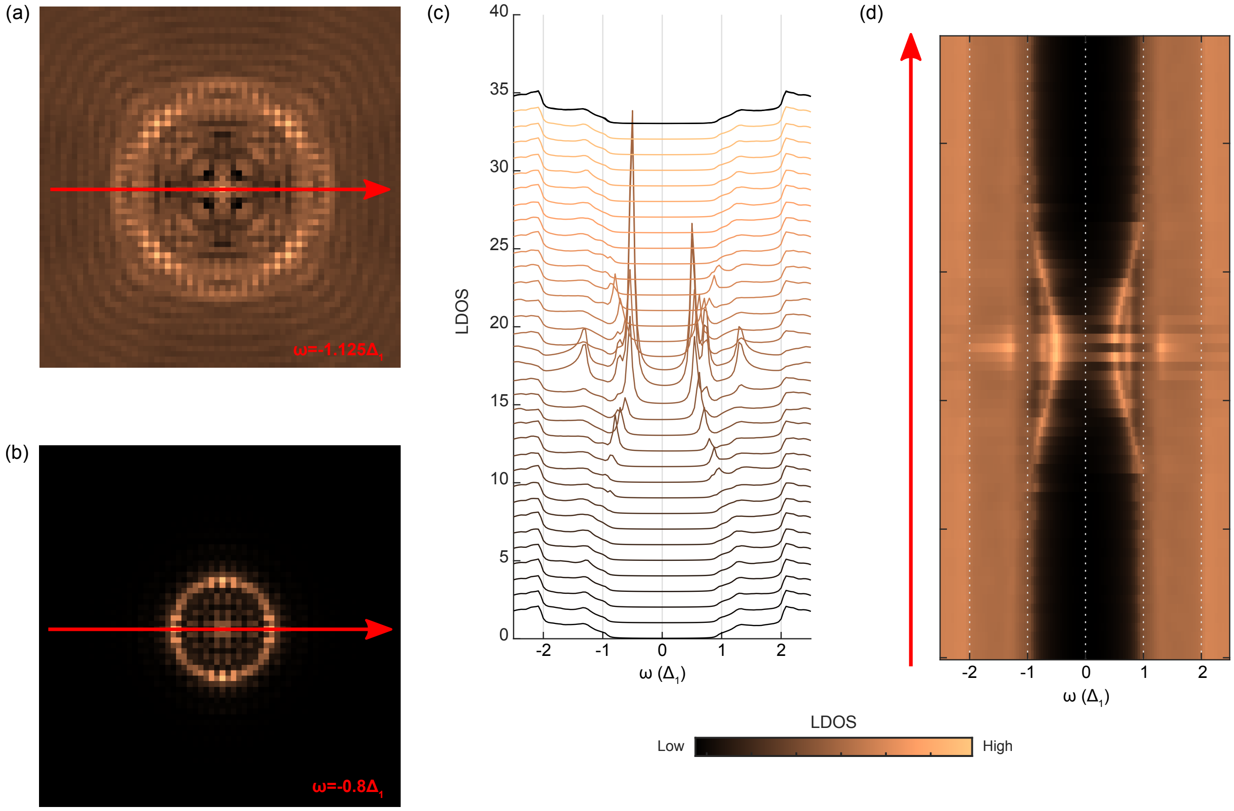

Figure S12: (a) and (b) Local density of states at two selected energies below and above Δ_1 for the strong impurity. Spectra along the cut indicated with an arrow showing the dispersive in-gap impurity-bound states ((c) and (d)) and the recovery of the homogeneous spectra (black spectra on top). All spectra are offset vertically by one unit, spectra plotted for every other lattice point.

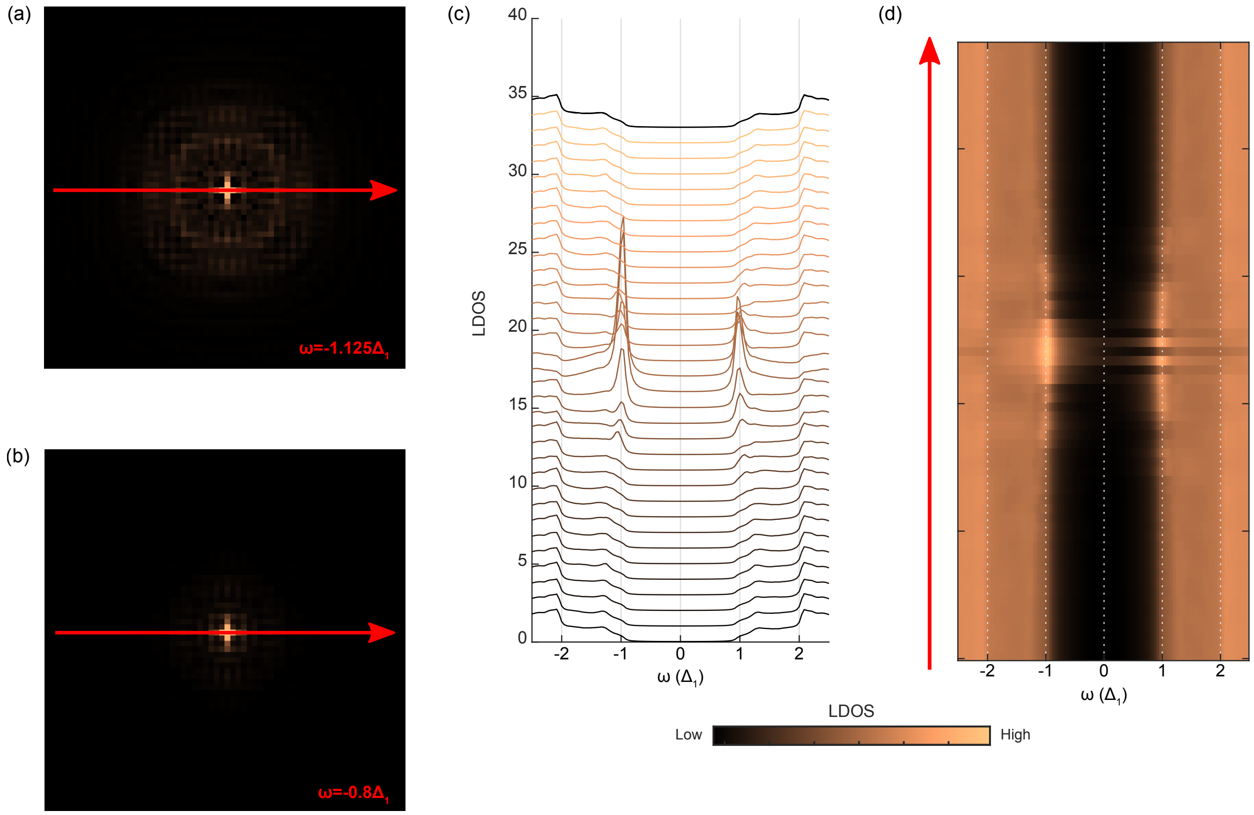

Figure S13: (a) and (b) Local density of states at two selected energies below and above Δ_1 for the weak impurity, were only a faint ring-like feature is visible at $|\omega| > \Delta_1$. Spectra along the cut indicated with an arrow showing the dispersive in-gap Shiba states as (c), waterfall plot and (d), intensity plot of the LDOS.

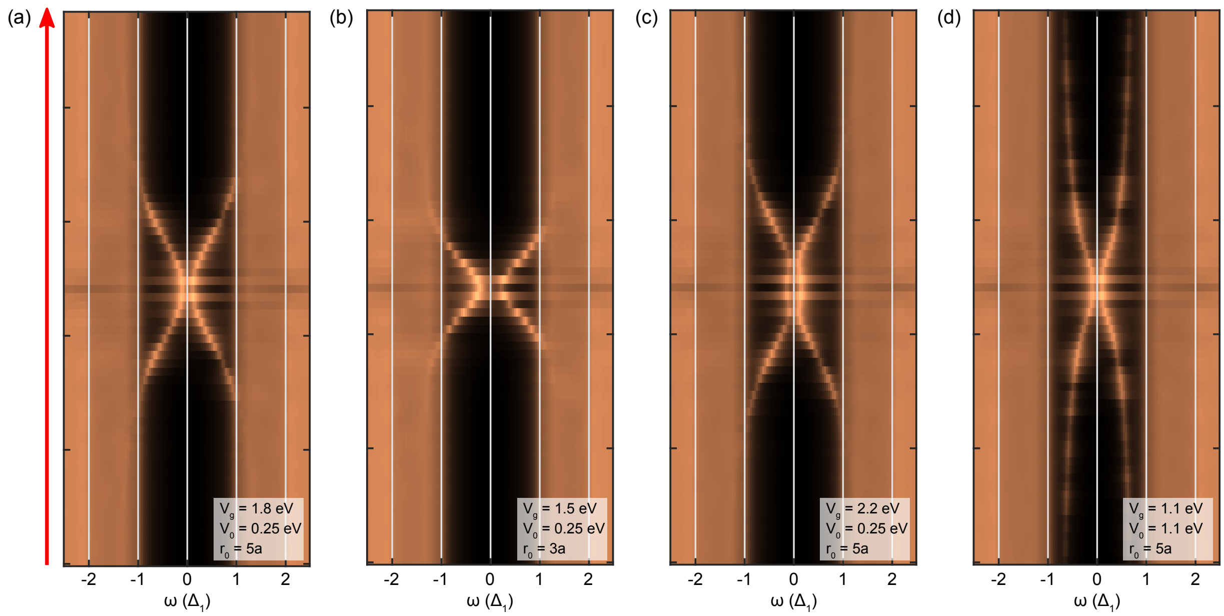

Figure S14: (a-d) Variations of in-gap states upon changing of the impurity strength V_r, the gating potential magnitude V_0 and the radial scale r_0, see labels on plots. All plots along the same path as in Figs. S12 and S13.

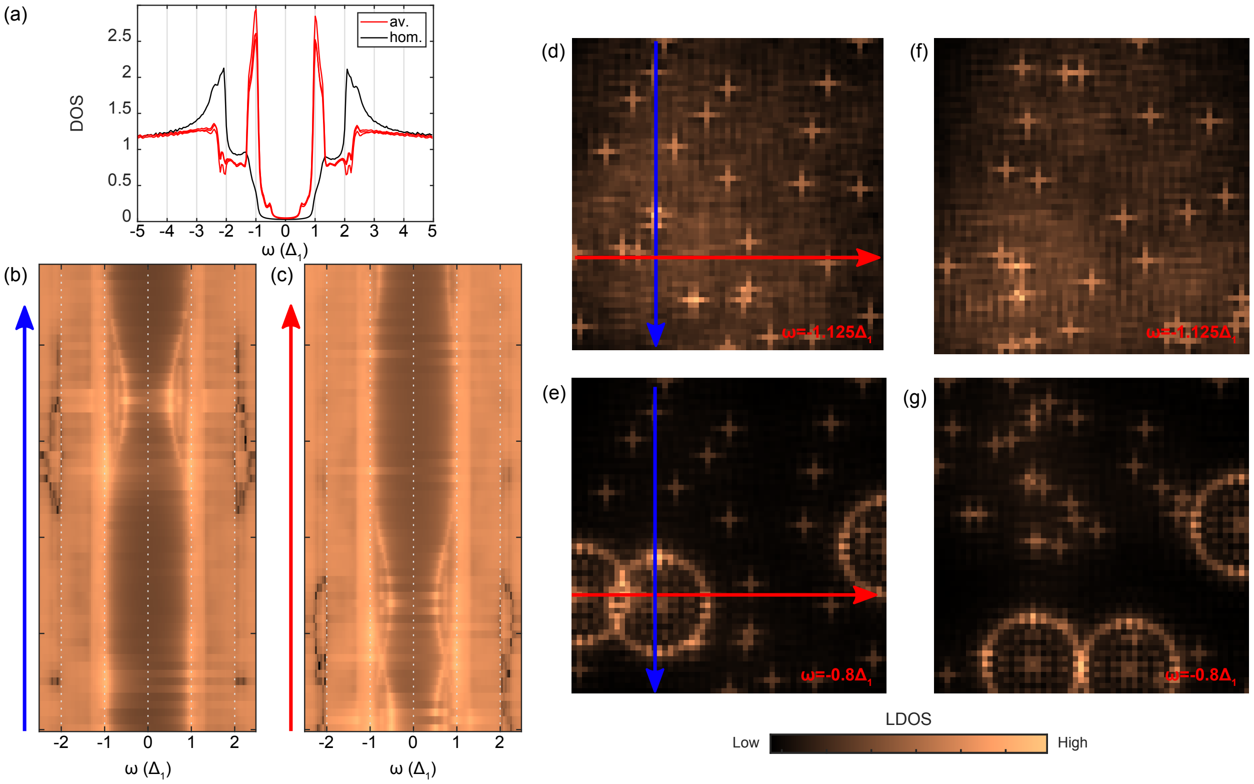

Figure S15: (a) Averaged spectra for different impurity configurations and different number of weak impurities $n = 30, 35$ in the field of view. (b) and (c) Cuts along colored arrows indicated in panels (d) and (e) for the LDOS maps at the two selective energies above and below Δ_1. (f) and (g) Another impurity configuration with $n = 35$ used to generate the variation of the averaged spectra in a.
